# Association of estimated glucose disposal rate with chronic kidney disease: comparative analysis against traditional insulin resistance indices

**DOI:** 10.3389/fendo.2025.1507735

**Published:** 2025-07-04

**Authors:** Yuanxin Liu, Mingda Liu, Yuyin Jiang, Siyuan Cui, Wei Tang

**Affiliations:** ^1^ Department of Endocrinology, Geriatric Hospital of Nanjing Medical University, Nanjing, Jiangsu, China; ^2^ The Core Laboratory, Nanjing BenQ Medical Center, The Affiliated BenQ Hospital of Nanjing Medical University, Nanjing, Jiangsu, China; ^3^ Wuxi People's Hospital, Nanjing Medical University, Wuxi, Jiangsu, China; ^4^ Wuxi Medical Center, The Affiliated Wuxi People's Hospital of Nanjing Medical University, Wuxi, Jiangsu, China

**Keywords:** eGDR, HOMA-IR, QUICKI, TyG, TyG-BMI, TyG-WC, eGFR, UACR

## Abstract

**Background:**

Chronic kidney disease (CKD) is a widespread condition, marked by significant morbidity and mortality rates, particularly in individuals with comorbidities such as diabetes and hypertension. While insulin resistance (IR) has been linked to CKD, the traditional methods used to measure IR have inherent limitations. This necessitates the exploration of alternative indicators that can more accurately reflect the relationship between IR and CKD.

**Methods:**

This study employed a cross-sectional design, utilizing data extracted from the National Health and Nutrition Examination Survey (NHANES) spanning the years 2013 to 2018. The study sample comprised 7423 participants. Comprehensive demographic, anthropometric, and laboratory data were collected and analyzed. The estimated glucose disposal rate (eGDR), along with established measures of insulin resistance such as HOMA-IR, QUICKI, and the TyG, TyG-BMI, and TyG-WC indices were computed. The relationships between these indices and CKD indicators, specifically the eGFR and UACR, were assessed using a combination of linear and logistic regression models. Additionally, the performance of these indices was evaluated using receiver operating characteristic (ROC) curve analysis.

**Results:**

Elevated levels of the eGDR were significantly correlated with improved kidney function and a reduced prevalence of chronic kidney disease (CKD) and albuminuria. The correlation coefficients (R²) demonstrated that eGDR had a stronger association with the estimated glomerular filtration rate (eGFR) at R²=0.1379 and with the urinary albumin-to-creatinine ratio (UACR) at R²=0.0816, compared to the traditional measures of insulin resistance. eGDR also declined progressively across worsening CKD stages (p for trend< 0.001), highlighting a dose–response relationship. Logistic regression analysis further revealed that higher eGDR levels were associated with a decreased risk of developing CKD and proteinuria. Additionally, the ROC curve analysis indicated that eGDR exhibited the highest predictive accuracy for CKD, with an area under the curve (AUC) of 0.75, and for proteinuria, with an area under the curve (AUC) of 0.68.

**Conclusion:**

The eGDR has emerged as a reliable and practical marker of insulin resistance associated with CKD indicators, demonstrating stronger associations with eGFR and UACR compared to traditional measures like HOMA-IR, QUICKI, TyG, TyG-BMI and TyG-WC. The simplicity of calculating eGDR enhances its utility as a valuable tool for the early detection and management of CKD, potentially improving clinical outcomes.

## Introduction

Chronic kidney disease (CKD) represents a significant global public health challenge, with an estimated prevalence approaching 10% among adults (1, 2). It is linked to considerable morbidity, mortality, and escalating healthcare expenditures. The asymptomatic progression of CKD to advanced stages underscores the critical need for the identification of reliable early biomarkers to facilitate timely intervention. CKD is hallmarked by alterations in kidney structure or function, commonly detected through a decline in the estimated glomerular filtration rate (eGFR) or the presence of increased albuminuria. The Kidney Disease: Improving Global Outcomes (KDIGO) guidelines offer a robust framework for the classification of CKD, categorized by GFR stages and albumin-to-creatinine ratio (ACR) categories. This framework is instrumental in evaluating kidney damage and monitoring disease progression (3).

CKD is notably prevalent among individuals with diabetes and hypertension (4). A complex interplay of factors contributes to CKD, encompassing sociodemographic, behavioral, genetic, cardiovascular, and metabolic elements (5). Among these, insulin resistance (IR) has emerged as a significant risk factor (6). IR is not only prevalent in the early stages of CKD (7) but also associated with various metabolic disturbances that can exacerbate kidney damage (8). Furthermore, insulin resistance is not confined to specific etiologies of kidney disease; it is observed in conditions such as diabetes, IgA nephropathy, and polycystic kidney disease (9).

Traditionally, insulin resistance (IR) is measured using the hyperinsulinemic-euglycemic clamp test (10), which is considered the gold standard. However, due to its complexity and cost, this method is impractical for routine clinical use. As a result, surrogate measures such as the Homeostasis Model Assessment of Insulin Resistance (HOMA-IR) and the Quantitative Insulin Sensitivity Check Index (QUICKI) are commonly employed. Despite their widespread use, these measures have inherent limitations, particularly in the context of patients with diabetes or those undergoing insulin therapy (6). Given that circulating insulin concentrations are rarely measured in primary care settings, alternative IR assessment markers such as the triglyceride-glucose (TyG) index have been developed (11). Additionally, the combination of the TyG index with obesity indices, such as TyG-BMI and TyG-WC, may offer superior predictive capability compared to the TyG index alone (12).However, few studies have evaluated the correlation between the TyG-related indices and prevalence in patients with CKD.

The estimated glucose disposal rate (eGDR) has emerged as a practical and reliable alternative for assessing IR. This marker comprehensively incorporates waist circumference, hypertension status, and glycated hemoglobin A1c (HbA1c) levels, thereby reflecting both central obesity and glucose metabolism. Its multifactorial nature renders eGDR particularly suitable for clinical practice and large-scale cohort studies (13). Although previous studies have validated eGDR in individuals with type 1 diabetes and demonstrated its association with a range of microvascular and macrovascular complications (14), the exploration of its relationship with kidney disease in other populations remains a relatively uncharted area of research.

This study is designed to investigate the correlation between the eGDR and indicators of CKD, specifically comparing its relationship with the eGFR and urinary albumin-to-creatinine ratio (UACR) to those of established insulin resistance measures, such as the HOMA-IR, the QUICKI and the TyG, TyG-BMI, and TyG-WC indices. Gaining insights into these associations may significantly improve the early detection and management strategies for CKD.

## Materials and methods

### Study design and population

This cross-sectional study leveraged data from the National Health and Nutrition Examination Survey (NHANES), encompassing a period from 2013 to 2018 and totaling 29,400 participants. NHANES, administered by the National Center for Health Statistics (NCHS) within the Centers for Disease Control and Prevention (CDC), encompasses a comprehensive set of components including physical examinations, health and nutrition questionnaires, and laboratory assessments. Upon extraction from the NHANES database, the dataset was refined to include only those participants, numbering 7,423, who had complete data available for the eGFR, UACR and insulin resistance measures, including the HOMA-IR, the QUICKI and the TyG, TyG-BMI, and TyG-WC indices, thus meeting the study's inclusion criteria.

### Data collection

Data collected from NHANES included demographic information (gender, age, education level, and annual household income), anthropometric measurements [waist circumference (WC) and body mass index (BMI)], and laboratory measurements [serum creatinine, fasting blood glucose (FBG), glycated hemoglobin, hemoglobin, fasting insulin, UACR, total cholesterol (TC), triglycerides (TG), high-density lipoprotein (HDL), low-density lipoprotein (LDL), blood urea nitrogen (BUN), uric acid (UA), aspartate transaminase (AST), alanine transaminase (ALT), and gamma-glutamyl transferase(GGT)].

### Variable calculations and definitions

eGDR was calculated using the formula (15):


eGDR (mg/kg/min) = 21.158 − (0.09 WC) − (3.407 hypertension) − (0.551 HbA1c) [WC (cm), hypertension (yes = 1/no = 0), and HbA1c (%)]

HOMA-IR was calculated using the formula (16):


$$HOM-IR=\frac{FastingInsulin(\mu U/mL)xFBG(mg/dL)}{405}$$

QUICKI was calculated using the formula (16):


$$QUICKI=\frac{1}{long(FastingInsulin)+\log(FBG)}$$

The TyG, TyG-BMI, and TyG-WC indices were calculated using the formulas (11, 17):


TyG index = Ln [fasting TG (mg/dL) × FBG (mg/dL)/2]

TyG−BMI index = TyG index × BMI

TyG−WC index = TyG index × WC

eGFR was calculated using the CKD-EPI equation, considering factors such as age, gender, ethnicity, and serum creatinine. CKD was defined as eGFR< 60 mL/min/1.73 m². Proteinuria was defined as UACR ≥ 30 mg/g, with microalbuminuria classified as UACR between 30–299 mg/g and macroalbuminuria as UACR ≥ 300 mg/g. Smoking, alcohol consumption and hypertension history were determined based on self-reported data. Diabetes mellitus (DM) was defined as FBG ≥ 7 mmol/L, HbA1c ≥ 6.5%, or self-reported history of diabetes. BMI was categorized as underweight (< 18.5), normal weight (18.5-25), overweight (25-29.9), and obese (≥30) kg/m².

### Statistical analysis

Multiple imputation using the predictive mean matching method handled missing data to minimize bias and make full use of available data. For missing data, we employed the mice package in R, utilizing Mean Matching (MMM) for imputation. We chose to generate one imputed dataset (m=1), with a predictor matrix defining the variables used for imputing missing data. The imputation covered several important covariates, including education status (701 missing values), smoking status (368 missing values), alcohol consumption (1,012 missing values), LDL-C (87 missing values), BUN (1 missing value), UA (2 missing values), AST (9 missing values), ALT (2 missing values), and GGT (2 missing values). We also performed sensitivity analyses to examine the robustness of our results to the missing data handling process. Continuous variables were expressed as means ± standard deviations (SD) for normally distributed data or medians and interquartile ranges (IQR) for non-normally distributed data. Categorical variables were expressed as frequencies and percentages. Comparisons between groups were performed using Student’s t-tests for normally distributed continuous variables, Mann-Whitney U tests for non-normally distributed continuous variables, and chi-square tests for categorical variables. Pearson and Spearman correlation analyses assessed the association of eGDR, HOMA-IR, QUICKI, TyG, TyG-BMI, TyG-WC indices with eGFR, and UACR. In addition, we categorized CKD by five eGFR-based stages and used linear regression to evaluate the trend of eGDR across CKD stages (p for trend).

Logistic regression models were employed to investigate the associations between the eGDR, HOMA-IR, QUICKI, TyG, TyG-BMI, and TyG-WC indices with the risk of CKD, defined as an eGFR less than 60 mL/min/1.73m^2^, and albuminuria, defined as a UACR greater than 30 mg/g. Three distinct models were utilized, each with varying degrees of covariate adjustment. Restricted Cubic Splines Smooth curve fitting analysis was used for CKD and albuminuria with eGDR, HOMA-IR, QUICKI, TyG, TyG-BMI, and TyG-WC indices as the independent variable. ROC analysis was used to evaluate the discriminatory performance of insulin sensitivity indices on CKD and albuminuria. The optimal cut-off points were identified based on the maximum Youden index. We further stratified the ROC analysis for eGDR by diabetes status and reported separate AUCs and cut-off values for diabetic and non-diabetic individuals. Subgroup analyses were performed based on age, gender, smoking status, alcohol consumption, BMI categories, hypertension, and diabetes status. Statistical analyses were performed using R 4.3.3 (http://www.R-project.org). A two-sided *P* value<0.05 was considered statistically significant.

## Results

### Study population and patient characteristics

The study included 7,423 participants with a mean age of 46.95 ± 19.04 years. Among them, 6.9% had chronic kidney disease (CKD). We compared general information and clinical indicators between the non-CKD and CKD groups (**Table 1**). The CKD group exhibited significantly higher values for age, BMI, WC, FBG, fasting insulin, HbA1c levels, TG, serum creatinine, BUN, UACR, UA, smoking history, and hypertension history compared to the non-CKD group (P< 0.05). Conversely, the CKD group had significantly lower values for educational level, annual household income (over $20,000), TC, LDL, hemoglobin. and eGFR (P< 0.05). Furthermore, the CKD group showed significantly lower eGDR values (5.72 ± 2.39 vs. 8.09 ± 2.75, P< 0.001), higher HOMA-IR values (2.96 [1.79, 5.22] vs. 2.41 [1.47, 4.20], P< 0.001), and lower QUICKI values (0.24 [0.21, 0.27] vs. 0.25 [0.22, 0.29], P< 0.001). Additionally, the CKD group showed significantly higher TyG index (8.68 [8.28, 9.15] vs. 8.43 [7.99, 8.91], P< 0.001), TyG-BMI (250.89 [214.10, 297.52] vs. 236.27 [195.92, 284.74], P< 0.001), and TyG-WC (900.33 [797.29, 1029.33] vs. 824.64 [699.29, 952.03], P< 0.001), indicating a poorer insulin sensitivity profile.

**Table 1:** Descriptive statistics for patients with and without CKD.

Characteristics	Overall (n=7423)	Non-CKD (n=6913)	CKD (n=510)	*P* value
eGDR, mg/kg/min	7.93 ± 2.80	8.09 ± 2.75	5.72 ± 2.39	<0.001
HOMA-IR	2.44 [1.48, 4.27]	2.41 [1.47, 4.20]	2.96 [1.79, 5.22]	<0.001
QUICKI	0.25 [0.22, 0.29]	0.25 [0.22, 0.29]	0.24 [0.21, 0.27]	<0.001
TyG,	8.45 [8.01, 8.93]	8.43 [7.99, 8.91]	8.68 [8.28, 9.15]	<0.001
TyG-BMI	237.77 [197.18, 285.76]	236.27 [195.92, 284.74]	250.89 [214.10, 297.52]	<0.001
TyG-WC	830.72 [704.95, 958.58]	824.64 [699.29, 952.03]	900.33 [797.29, 1029.33]	<0.001
Age, years	46.95 ± 19.04	45.14 ± 18.26	71.56 ± 10.27	<0.001
Female, n (%)	3813 (51.4)	3562 (51.5)	251 (49.2)	0.336
Education, n (%)				0.015
Less than 9th grade	656 (8.8)	603 (8.7)	53 (10.4)	
9-11th grade	966 (13.0)	893 (12.9)	73 (14.3)	
High school graduate	1662 (22.4)	1526 (22.1)	136 (26.7)	
Some college or AA degree	2283 (30.8)	2140 (31.0)	143 (28.0)	
College graduate or above	1856 (25.0)	1751 (25.3)	105 (20.6)	
Income, n (%)				<0.001
Over $20,000	6068 (81.7)	5689 (82.3)	379 (74.3)	
Under $20,000	1355 (18.3)	1224 (17.7)	131 (25.7)	
BMI, kg/m^2^	27.90 [24.00, 32.70]	27.80 [23.90, 32.70]	29.00 [25.30, 32.90]	<0.001
BMI category, n (%)				<0.001
Underweight	141 (1.9)	137 (2.0)	4 (0.8)	
Normal weight	2143 (28.9)	2032 (29.4)	111 (21.8)	
Overweight	2316 (31.2)	2142 (31.0)	174 (34.1)	
Obesity	2823 (38.0)	2602 (37.6)	221 (43.3)	
WC, cm	98.70 ± 17.20	98.22 ± 17.21	105.19 ± 15.57	<0.001
FBG mmol/L	5.61 [5.22, 6.16]	5.55 [5.22, 6.11]	6.05 [5.48, 7.05]	<0.001
Fasting insulin, μU/mL	9.51 [6.08, 15.45]	9.41 [6.01, 15.35]	10.92 [6.71, 16.83]	<0.001
HbA1C, %	5.50 [5.20, 5.90]	5.50 [5.20, 5.80]	5.90 [5.60, 6.57]	<0.001
Hemoglobin g/dL	14.10 [13.20, 15.10]	14.20[13.20, 15.20]	13.50[12.30, 14.57]	<0.001
ALT, U/L	19.00 [15.00, 27.00]	20.00 [15.00, 27.00]	17.00 [14.00, 23.00]	<0.001
AST, U/L	21.00 [18.00, 26.00]	21.00 [18.00, 26.00]	21.50 [18.00, 26.00]	0.918
GGT, U/L	19.00 [15.00, 27.00]	20.00 [15.00, 27.00]	17.00 [14.00, 23.00]	<0.001
TC, mmol/L	4.71 [4.03, 5.43]	4.71 [4.06, 5.43]	4.50 [3.85, 5.43]	0.001
TG, mmol/L	1.02 [0.69, 1.53]	1.00 [0.68, 1.51]	1.20 [0.86, 1.68]	<0.001
HDL, mmol/L	1.32 [1.11, 1.63]	1.34 [1.11, 1.63]	1.29 [1.09, 1.65]	0.209
LDL, mmol/L	2.74 [2.17, 3.39]	2.77 [2.20, 3.39]	2.51 [1.97, 3.21]	<0.001
BUN, mmol/L	4.64 [3.57, 5.71]	4.64 [3.57, 5.71]	7.85 [6.43, 10.00]	<0.001
Serum Creatinine, mg/dL	0.83 [0.69, 0.99]	0.81 [0.68, 0.94]	1.32 [1.16, 1.56]	<0.001
eGFR, mL/min/1.73m²	97.81 ± 24.49	101.59 ± 20.64	46.49 ± 11.08	<0.001
UACR, mg/g	7.34 [4.73, 14.07]	7.08 [4.64, 13.07]	15.80 [7.80, 79.44]	<0.001
UA, µmol/L	315.20 [261.70, 380.70]	315.20 [261.70, 368.80]	392.60 [327.10, 458.00]	<0.001
Smoking, %	3047 (41.0)	2787 (40.3)	260 (51.0)	<0.001
Drinking, %	5821 (78.4)	5432 (78.6)	389 (76.3)	0.245
Hypertension, %	2540 (34.2)	2164(31.3)	376 (73.7)	<0.001
Diabetes, %	1337 (18.0)	1129 (16.3)	208 (40.8)	<0.001
Albuminuria, %	900 (12.1)	709 (10.3)	191 (37.5)	<0.001

### eGDR tertile analysis

Participants were divided into three groups based on their eGDR levels: Tertile 1 (low eGDR), Tertile 2 (middle eGDR), and Tertile 3 (high eGDR) (**Table 2**). Compared to the low eGDR group, the middle and high eGDR groups had significantly lower values for age, BMI, waist circumference, HbA1c, TG, serum creatinine, UACR, serum uric acid, fasting blood glucose, fasting insulin, smoking history, diabetes, proteinuria, hypertension, and CKD prevalence, concurrently, they had significantly higher incomes and eGFR (P< 0.05). Additionally, significant differences were observed in educational level, gender distribution, TC, HDL-C, and LDL-C (P< 0.05).

**Table 2:** Descriptive statistics for patients by eGDR tertile.

Characteristics	Tertile 1 (n=2475)	Tertile 2 (n=2474)	Tertile 3 (n=2474)	*P* value
eGDR, mg/kg/min	4.59 ± 1.55	8.38 ± 0.89	10.82 ± 0.72	<0.001
HOMA-IR	3.81 [2.34, 6.71]	2.72 [1.74, 4.34]	1.55 [1.03, 2.24]	<0.001
QUICKI	0.22 [0.20, 0.25]	0.24 [0.22, 0.27]	0.28 [0.25, 0.32]	<0.001
TyG,	8.77 [8.31, 9.22]	8.53 [8.12, 8.95]	8.08 [7.72, 8.47]	<0.001
TyG-BMI	280.88 [243.13, 338.21]	255.31 [224.95, 288.94]	187.37 [167.09, 210.80]	<0.001
TyG-WC	960.38 [856.47, 1095.47]	873.27 [795.12, 956.03]	669.59 [601.69, 733.51]	<0.001
Age, years	57.58 ± 15.58	47.48 ± 17.95	35.79 ± 16.87	<0.001
Female, n (%)	1240 (50.1)	1189 (48.1)	1384 (55.9)	<0.001
Education, n (%)				<0.001
Less than 9th grade	249 (10.1)	249 (10.1)	158 (6.4)	
9-11th grade	339 (10.1)	329 (13.3)	298 (12.0)	
High school graduate	597 (24.1)	562 (22.7)	503 (20.3)	
Some college or AA degree	795 (32.1)	771 (31.2)	717 (29.0)	
College graduate or above	495 (20.0)	563 (22.8)	798 (32.3)	
Income, n (%)				<0.001
Over $20,000	1937 (78.3)	2052 (82.9)	2079 (84.0)	
Under $20,000	538 (21.7)	422 (17.1)	395 (16.0)	
BMI, kg/m^2^	31.80 [27.90, 37.50]	29.70 [26.60, 33.40]	23.30 [21.10, 25.50]	<0.001
BMI category, n (%)				<0.001
Underweight	0 (0.0)	17 (0.7)	124 (5.0)	
Normal weight	176 (7.1)	376 (15.2)	1591 (64.3)	
Overweight	761 (30.7)	876 (35.4)	679 (27.4)	
Obesity	1538 (62.1)	1205 (48.7)	80 (3.2)	
WC, cm	111.73 ± 15.89	101.89 ± 11.47	82.47 ± 7.72	<0.001
FBG, mmol/L	6.11 [5.55, 7.22]	5.61 [5.27, 6.11]	5.33 [5.00, 5.61]	<0.001
Fasting insulin, μU/mL	13.13 [8.38, 21.50]	10.66 [6.93, 16.36]	6.54 [4.42, 9.46]	<0.001
HbA1C, %	5.90 [5.50, 6.60]	5.50 [5.30, 5.80]	5.30 [5.10, 5.50]	<0.001
Hemoglobin, g/dL	14.00 [13.10, 15.10]	14.20[13.20, 15.30]	14.10[13.20, 15.10]	<0.001
ALT, U/L	21.00 [16.00, 30.00]	21.00 [16.00, 29.00]	17.00 [13.00, 22.00]	<0.001
AST, U/L	22.00 [18.00, 27.00]	22.00 [18.00, 27.00]	21.00 [18.00, 25.00]	<0.001
GGT, U/L	21.00 [16.00, 30.00]	21.00 [16.00, 29.00]	17.00 [13.00, 22.00]	<0.001
TC, mmol/L	4.71 [4.03, 5.51]	4.89 [4.24, 5.53]	4.50 [3.88, 5.25]	<0.001
TG, mmol/L	1.23 [0.85, 1.77]	1.11 [0.76, 1.65]	0.76 [0.54, 1.12]	<0.001
HDL, mmol/L	1.24 [1.06, 1.50]	1.27 [1.06, 1.55]	1.47 [1.24, 1.78]	<0.001
LDL, mmol/L	2.74 [2.15, 3.41]	2.92 [2.35, 3.54]	2.59 [2.04, 3.18]	<0.001
BUN, mmol/L	5.00 [3.93, 6.43]	4.64 [3.57, 5.71]	4.28 [3.57, 5.36]	<0.001
Serum Creatinine, mg/dL	0.87 [0.72, 1.05]	0.83 [0.70, 0.99]	0.79 [0.67, 0.93]	<0.001
eGFR, mL/min/1.73m²	86.97 ± 24.74	97.58 ± 22.78	108.88 ± 20.70	<0.001
UACR, mg/g	10.20 [5.88, 25.00]	6.74 [4.40, 12.13]	6.27 [4.38, 10.53]	<0.001
UA, µmol/L	345.00 [285.50, 410.40]	327.10 [273.60, 380.70]	285.50 [237.90, 339.00]	<0.001
Smoking, %	1220 (49.3)	1056 (42.7)	771 (31.2)	<0.001
Drinking, %	1924 (77.7)	1981 (80.1)	1916 (77.4)	0.046
Hypertension, %	2175 (87.9)	365 (14.8)	0 (0.0)	<0.001
Diabetes, %	961 (38.8)	321 (13.0)	55 (2.2)	<0.001
Albuminuria, %	543 (21.9)	215 (8.7)	142 (5.7)	<0.001
CKD, %	343 (13.9)	137 (5.5)	30 (1.2)	<0.001

With the increase in eGDR levels, a marked improvement in clinical outcomes related to kidney health was observed. The eGFR demonstrated a significant upward trend across increasing eGDR tertiles, with values of 86.97 ± 24.74, 97.58 ± 22.78, and 108.88 ± 20.70, respectively (all P< 0.001). In contrast, the UACR exhibited a significant downward trend, with values decreasing from 10.20 [5.88, 25.00] to 6.74 [4.40, 12.13], and further to 6.27 [4.38, 10.53] across the same tertiles (all P< 0.001).

**Figure 1:**
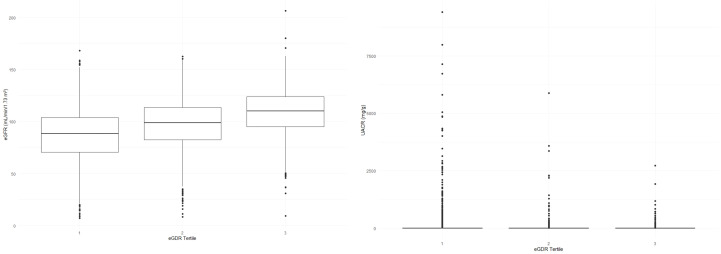
eGFR & UACR distribution across eGDR tertiles.

Additionally, the prevalence of albuminuria and CKD followed a significant decreasing trend with higher eGDR levels, dropping from 21.9% to 8.7%, and finally to 5.8% for albuminuria, and from 13.9% to 5.5%, and to 1.2% for CKD (all P< 0.001). The distribution of eGFR and UACR across the eGDR tertiles, which underscores these significant differences and trends, is depicted in **Figure 1**. Collectively, these findings suggest that higher eGDR levels are linked to improved kidney function and a reduced prevalence of CKD and albuminuria.

### Correlations between insulin sensitivity indices (eGDR, HOMA-IR, QUICKI, TyG, TyG-BMI, TyG-WC) and kidney function markers (eGFR, UACR)

We demonstrated a significant positive correlation between eGDR and eGFR (**Table 3**). Notably, eGDR exhibited the highest coefficient of determination (R²) value of 0.1370 among the indices studied, suggesting a relatively stronger association with eGFR within the chronic kidney disease (CKD) group. However, it is important to note that the R² values for all six indices, while statistically significant, did not indicate particularly strong relationships.

**Table 3:** Linear regression between insulin sensitivity indices (eGDR, HOMA-IR, QUICKI, TyG, TyG-BMI, TyG-WC) and eGFR.

Index	Estimate (β)	Std. Error	*t* value	*P* value	R²	Adjusted R²
eGDR	3.24263	0.09447	34.65	<0.001	0.1370	0.1369
HOMA-IR	-0.20219	0.03379	-5.983	<0.001	0.0048	0.0047
QUICKI	10.2138	3.3209	3.076	<0.001	0.0013	0.0011
TyG	-7.9102	0.3909	-20.23	<0.001	0.0523	0.0522
TyG-BMI	-0.041554	0.004071	-10.21	<0.001	0.0138	0.0137
TyG-WC	-0.031802	0.001497	-21.25	<0.001	0.0574	0.0572

Further analysis (**Table 4**) demonstrated a significant negative correlation between eGDR and log(UACR+1). The eGDR had the highest R² value (0.0813). Similar to the findings for eGFR, eGDR had the highest R² among the three indices; however, all R² values were relatively low modest, indicating weak linear correlations with UACR. These results suggest that the relationships between insulin sensitivity indices and kidney function markers may not be straightforward and could involve more complex, potentially non-linear dynamics.

**Table 4:** Linear regression analysis between insulin sensitivity indices (eGDR, HOMA-IR, QUICKI) and log(UACR + 1).

Index	Estimate (β)	Std. Error	*t* value	*P* value	R²	Adjusted R²
eGDR	-0.108291	0.004223	-25.64	<0.001	0.0814	0.0813
HOMA-IR	0.022910	0.001443	15.87	<0.001	0.0328	0.0327
QUICKI	-0.77123	0.14369	-5.37	<0.001	0.0038	0.0037
TyG	0.30322	0.01704	17.797	<0.001	0.0409	0.0408
TyG-BMI	0.0019225	0.0001762	-10.91	<0.001	0.0158	0.0157
TyG-WC	0.0009838	0.0000658	-14.95	<0.001	0.0292	0.0291

**Figure 2:**
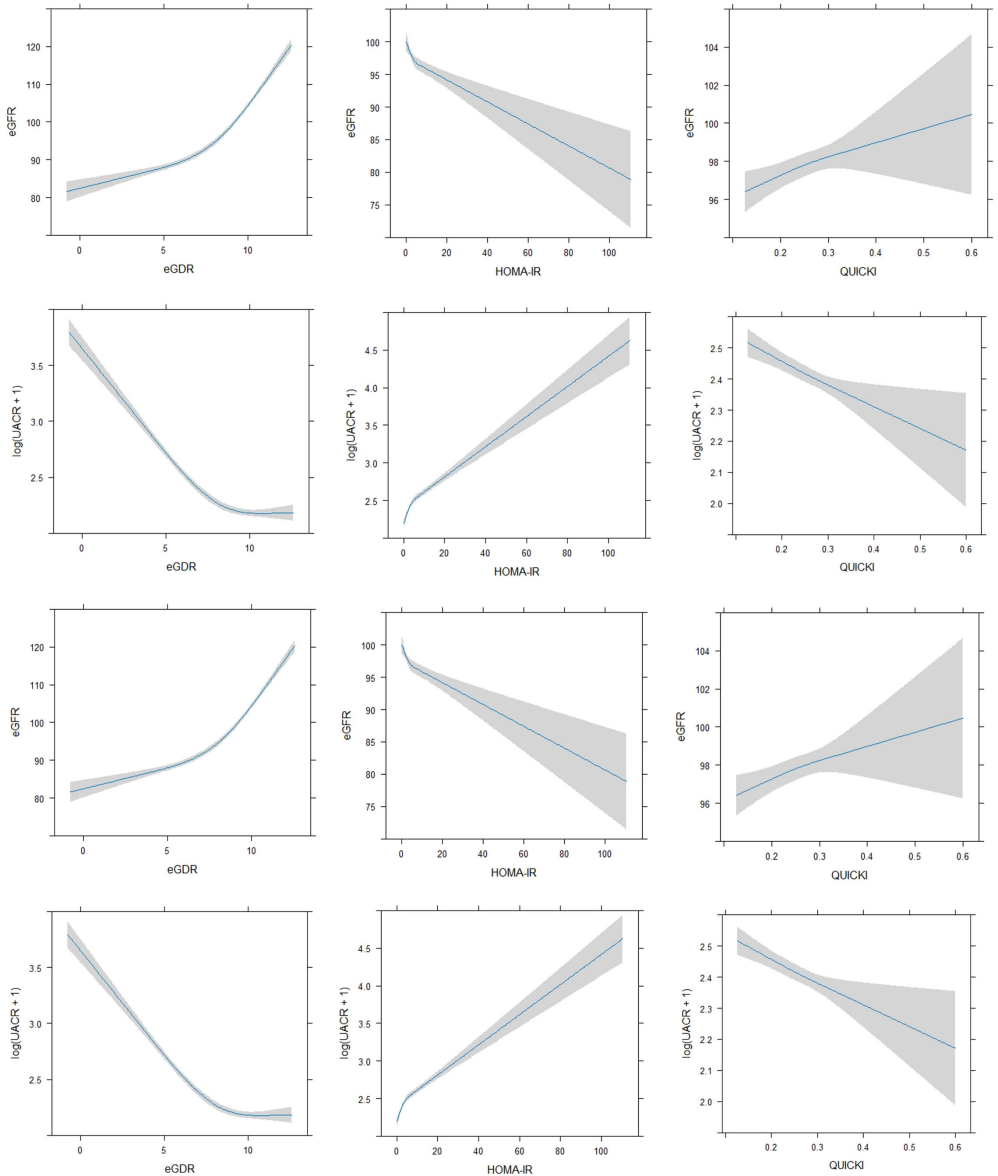
Non-linear regression between insulin sensitivity indices (eGDR, HOMA-IR, QUICKI) and kidney function markers (eGFR, UACR).

Given these findings, we further explored the relationships using Restricted Cubic Splines (RCS) to assess potential non-linear associations (**Figure 2**). This analytical approach allowed for a more refined comprehension of the intricate interactions between insulin sensitivity indices and kidney function markers, revealing nuances that may not be apparent with linear analysis.

To further explore the relationship between eGDR and CKD severity, we evaluated the distribution of eGDR levels across CKD stages (G1–G5) as classified by eGFR (**Table 5**). A linear regression model treating CKD stage as an ordinal variable demonstrated a significant decreasing trend in eGDR levels with advancing CKD stage (p for trend< 0.001). This finding reinforces the observed negative association between eGDR and eGFR, suggesting that insulin sensitivity, as measured by eGDR, progressively declines as kidney function deteriorates.

**Table 5:** Association between eGDR and CKD stages based on eGFR classification.

Variable	CKD 1	CKD 2	CKD 3	CKD 4	CKD 5	P for trend
**eGDR** mg/kg/min	7.53 ± 2.69	7.12 ± 2.65	5.79 ± 2.36	4.58 ± 2.25	6.34 ± 3.09	<0.001

### Logistic regression analysis of CKD and albuminuria

We performed logistic regression analysis to investigate the associations between insulin sensitivity indices (eGDR, HOMA-IR, QUICKI, TyG, TyG-BMI, TyG-WC) and the risk of CKD. The results, summarized in **Table 6**, indicated that higher eGDR levels were significantly associated with a lower risk of CKD across all models. Specifically, in Model 3, the odds ratio (OR) for eGDR was 0.879 (95% CI: 0.820 – 0.941, P<0.001). Conversely, higher HOMA-IR levels were associated with an increased risk of CKD, though this association reaching nominal significance in Model 3 (OR: 1.010, 95% CI: 1.010 – 1.020, P = 0.004). QUICKI and TyG, TyG-BMI, TyG-WC indices did not show significant associations with CKD after adjustment for additional variables.

**Table 6:** Logistic regression analysis for CKD.

Model	OR	95% CI	*P* value
eGDR			
Model 1	0.754	0.730 - 0.778	<0.001
Model 2	0.836	0.802 - 0.871	<0.001
Model 3	0.87	0.812 - 0.931	<0.001
HOMA-IR			
Model 1	1.02	1.014 - 1.027	<0.001
Model 2	1.018	1.011 - 1.025	<0.001
Model 3	1.014	1.006 - 1.024	0.002
QUICKI			
Model 1	0.378	0.158 - 0.779	0.013
Model 2	0.297	0.133 - 0.676	0.001
Model 3	0.587	0.232 – 2.692	0.334
TyG			
Model 1	1.562	1.386 – 1.757	<0.001
Model 2	1.305	1.116 – 1.524	<0.001
Model 3	0.95	0.605 – 1.590	0.836
TyG-BMI			
Model 1	1.003	1.002 – 1.004	<0.001
Model 2	1.004	1.003 - 1.006	<0.001
Model 3	1.006	0.991 – 1.021	0.464
TyG-WC			
Model 1	1.002	1.002 – 1.003	<0.001
Model 2	1.002	1.001 – 1.002	<0.001
Model 3	1.001	0.999 – 1.003	0.156

OR, odds ratio.

95% CI, 95% confidence interval.

Model 1: unadjusted model;

Model 2: age, gender, and ethnicity were adjusted.

Model 3: additionally adjusted for annual household income, education level, smokers, drinking, BMI, ALT, AST, GGT, FBG, HbA1c, hemoglobin, TG, TC, HDL-c, LDL-c, and SUA.

We also evaluated the associations between these indices and albuminuria, as summarized in **Table 7**. Higher eGDR levels were significantly associated with a lower risk of albuminuria in all models, with Model 3 showing an OR of 0.852 (95% CI: 0.813 - 0.893, P< 0.001). HOMA-IR and QUICKI, TyG, TyG-BMI, TyG-WC indices, however, showed no significant associations with albuminuria in Model 3 after adjustment for other variables.

**Table 7:** Logistic regression analysis for albuminuria.

Model	OR	95% CI	*P* value
eGDR			
Model 1	0.788	0.769 - 0.808	<0.001
Model 2	0.824	0.802 - 0.847	<0.001
Model 3	0.85	0.811 - 0.890	<0.001
HOMA-IR			
Model 1	1.034	1.027 - 1.042	<0.001
Model 2	1.03	1.023 - 1.039	<0.001
Model 3	1.008	1.000 - 1.016	0.046
QUICKI			
Model 1	0.172	0.0534 - 0.431	<0.001
Model 2	0.26	0.098 - 0.566	0.003
Model 3	1.637	0.566 - 6.715	0.472
TyG			
Model 1	1.883	1.713 – 2.070	<0.001
Model 2	1.702	1.538 – 1.882	<0.001
Model 3	0.988	0.771 – 1.257	0.92
TyG-BMI			
Model 1	1.004	1.004 - 1.005	<0.001
Model 2	1.004	1.003 - 1.005	<0.001
Model 3	1.005	0.998 - 1.013	0.17
TyG-WC			
Model 1	1.002	1.002 – 1.003	<0.001
Model 2	0.297	0.133 - 0.676	0.001
Model 3	1	0.999 – 1.001	0.74

OR, odds ratio.

95% CI, 95% confidence interval.

Model 1: unadjusted model;

Model 2: age, gender, and ethnicity were adjusted.

Model 3: additionally adjusted for annual household income, education level, smokers, drinking, BMI, ALT, AST, GGT, FBG, HbA1c, hemoglobin, TG, TC, HDL-c, LDL-c, and SUA.

**Figure 3:**
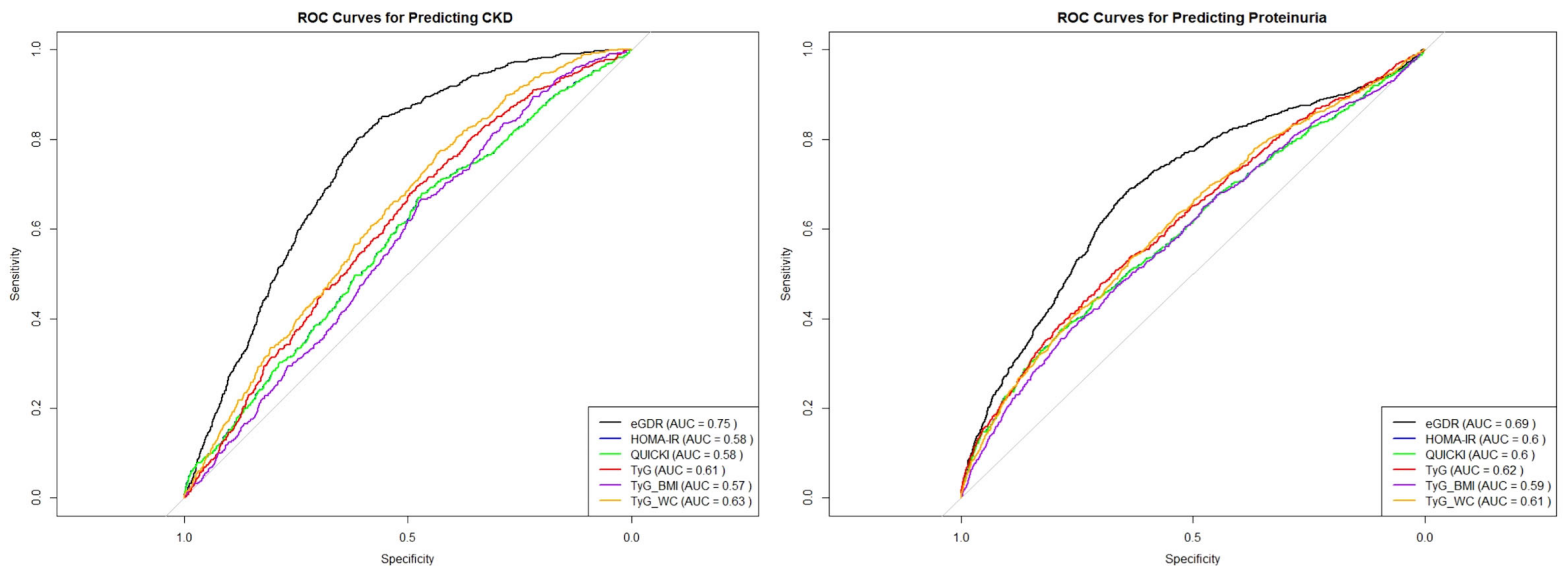
ROC curve analysis for eGDR, HOMA-IR, QUICKI and TyG-related indices.

To further assess the predictive power of these insulin sensitivity indices for CKD and proteinuria, we conducted Receiver Operating Characteristic (ROC) curve analysis for eGDR, HOMA-IR, QUICKI and TyG related indices as presented in **Figure 3**. The analysis revealed that eGDR had the highest area under the curve (AUC) value of 0.75, indicating it as the most potent predictor for CKD among the three indices. The optimal cut-off point determined from the ROC analysis was 7.7393 for CKD. In comparison, both HOMA-IR and QUICKI had AUC values of 0.57, demonstrating lower predictive accuracy. The TyG index also demonstrated a moderate predictive power with an AUC of 0.61, while TyG-BMI and TyG-WC had AUC values of 0.57 and 0.63, respectively. In further stratified analyses, eGDR demonstrated an AUC of 0.59 in individuals with diabetes and 0.75 in those without diabetes. Correspondingly, the optimal cut-off values for predicting CKD were 5.56 for the diabetic group and 8.98 for the non-diabetic group (**Supplementary Figure S1**).

In terms of predicting proteinuria, the ROC curve analysis indicated that eGDR again outperformed the other indices, with an AUC of 0.69, and the best cut-off value of eGDR was 7.864. The TyG-WC index followed with an AUC of 0.61, while TyG showed an AUC of 0.62, demonstrating a slightly better predictive power compared to QUICKI, which had an AUC of 0.60. HOMA-IR and TyG-BMI demonstrated the lowest predictive values among the indices for proteinuria with AUCs of 0.60 and 0.59, respectively.

In summary, these results confirm that eGDR is not only a strong indicator of CKD and proteinuria risk but also the most reliable predictor when compared to HOMA-IR, QUICKI, and TyG, TyG-BMI, TyG-WC indices among the six insulin sensitivity indices.

### Subgroup analysis

**Figure 4:**
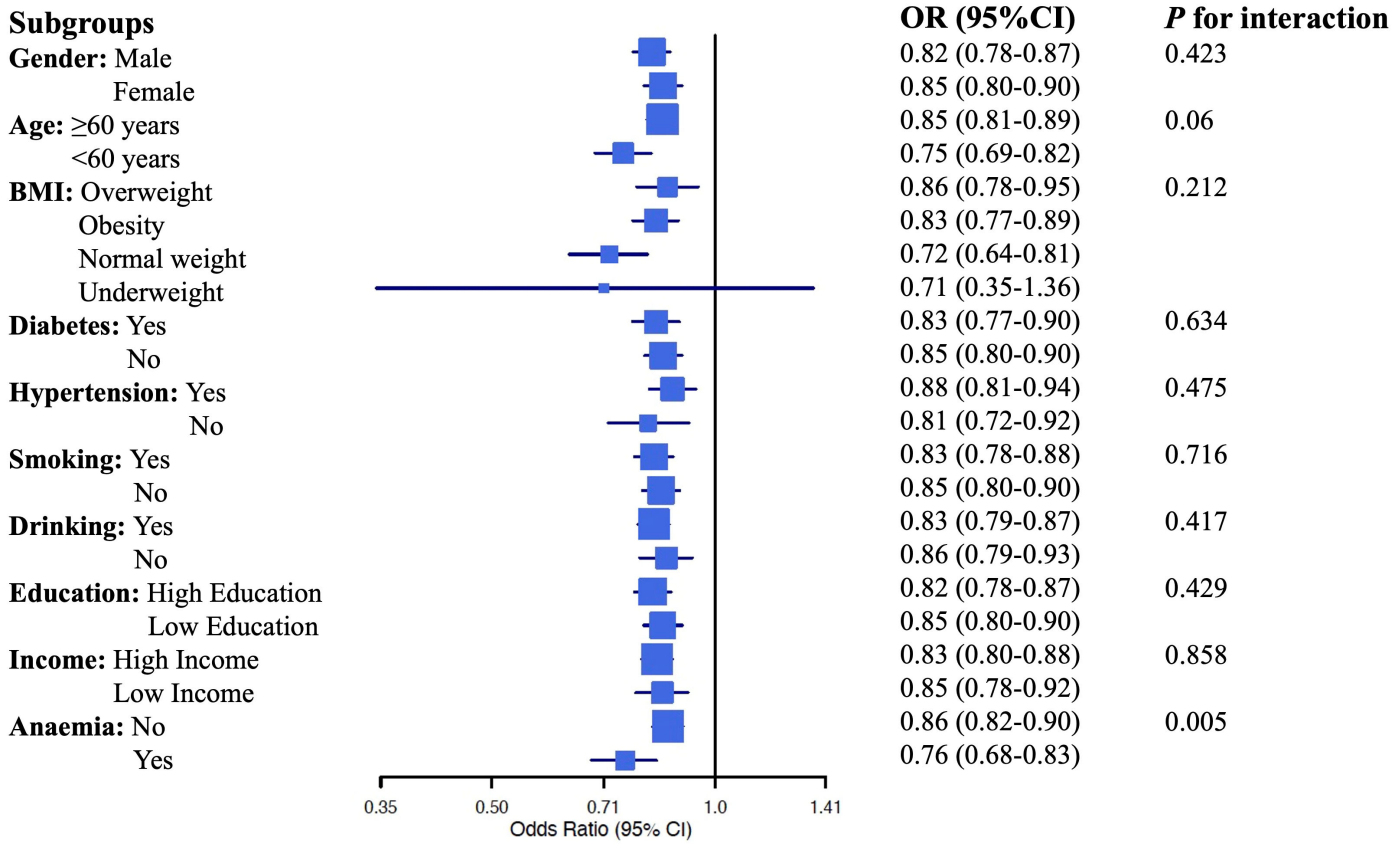
Forrest plot of dubgroup analysis.

We conducted a subgroup analysis to explore the predictive power of eGDR across different subpopulations based on gender, age, BMI categories, presence of diabetes, smoking, and drinking history, education level, and income status. The results are summarized in the forest plot (**Figure 4**). The subgroup analysis revealed that higher eGDR consistently remained a significant predictor of CKD across most subgroups, with particularly strong associations observed among individuals under the age of 60, those with normal weight, non-smokers, and non-drinkers. However, eGDR's predictive power was less significant among underweight individuals. Importantly, the predictive association between eGDR and CKD also differed by anemia status. We observed a statistically significant interaction (p for interaction = 0.005), suggesting that anemia may modify the association between eGDR and CKD. The p for interaction values in this analysis provide insights into whether the predictive power of eGDR differs significantly across the various subgroups. In this case, except for anemia, the lack of statistically significant p for interaction values suggests that the predictive capability of eGDR is generally stable across different subgroups. Overall, this comprehensive subgroup analysis underscores eGDR's robust predictive capability for CKD across diverse demographic and clinical profiles.

## Discussion

Our findings confirm that insulin resistance and hyperinsulinemia are strongly associated with CKD, consistent with previous studies (18). IR appears early in CKD progression and remains prevalent across various stages of kidney disease (9), including the initial stage (19). In a previous small sample study using the hyperinsulinemic euglycemic glucose clamp technique, it was found that the glucose disposal rate (GDR) was 9.93 ± 1.33 mg/kg/min individuals without CKD and 6.91 ± 2.46 mg/kg/min in CKD patients (20). Additionally, HOMA-IR, a common index for assessing insulin resistance, has been associated with eGFR levels in non-diabetic middle-aged U.S. adults (21).

Similarly, the Health Aging and Body Composition study showed an inverse relationship between HOMA-IR and eGFR in older individuals without diabetes at baseline (22). Our study corroborated these results, revealing that HOMA-IR was negatively correlated with eGFR and positively correlated with the incidence of CKD. However, some studies suggest that changes in eGFR are unrelated to IR (23), possibly due to differences in study design, population characteristics, or methods for assessing insulin resistance. To address these inconsistencies, we extended our analysis beyond traditional IR indices and investigated the association of eGDR with kidney function. Our study adds to this body of literature by demonstrating a significant, yet modest, relationship between eGDR and CKD markers. This suggests that while IR is an important factor, other variables, including metabolic health and comorbid conditions, may contribute to CKD risk. Future research should aim to resolve these discrepancies by using standardized methods and considering a broader range of confounders. We then evaluated the insulin sensitivity check index QUICKI for its association with CKD. Previous evaluations of QUICKI and CKD were not found, but we observed statistically significant, albeit weak, correlations with both eGFR and the UACR. After adjusting for multiple variables in logistic regression, QUICKI was not statistically significant for CKD prevalence. Another study reported a significant association between IR and eGFR, but this association disappeared after adjusting for variables, including BMI (24). These findings suggest that the relationship between insulin resistance and kidney function may be modulated by BMI, particularly in patients with normal kidney function.

The TyG index has gained increasing attention as a simple and accessible marker of insulin resistance, particularly in comparison to traditional indices like HOMA-IR and QUICKI (25). TyG-related indices, particularly in TyG-BMI and TyG-WC indices were significantly associated with the all-cause mortality, cardiovascular mortality, and diabetes mortality (17). Moreover, studies proved that individuals with long-term exposure to high TyG index levels had a significantly greater risk of CKD (26). TyG's incorporation of both fasting glucose and triglyceride levels makes it more sensitive to subtle metabolic changes. However, this sensitivity may also make TyG less stable, as it is more susceptible to external factors such as dietary intake and physiological state at the time of measurement. This variability could explain why, in our study, TyG showed a modest association with CKD indicators but did not demonstrate the robust predictive power observed with eGDR.

In recent years, eGDR has emerged as a proxy for insulin resistance, showing good correlation with IR and has been validated for estimating insulin sensitivity in individuals with type 1 diabetes​ (27). It has been associated with macrovascular events and mortality in type 1 diabetes patients, as well as microvascular complications such as retinopathy and nephropathy in young T1D patients​ (28). Its association extends to type 2 diabetes patients, where eGDR has been linked to the prevalence and severity of retinopathy (29). Regarding renal outcomes, eGDR has been associated with declining eGFR levels and higher incidence rates of eGFR<60 mL/min/1.73 m² or kidney function deterioration events (30). Furthermore, eGDR is related to the risk of incident cardiovascular diseases in non-diabetic individuals (15), suggesting its potential applicability to a broader patient population. Consistent with these findings, our study observed that eGDR is significantly associated with CKD indicators such as eGFR and UACR.

The significant associations between eGDR and CKD indicators such as eGFR and UACR suggest that eGDR is a robust marker of IR in the context of CKD. eGDR's components—waist circumference, hypertension, and HbA1c—capture essential aspects of metabolic health that are crucial in CKD pathophysiology. Central obesity, as measured by waist circumference, is a key driver of IR and associated metabolic disturbances (31). Hypertension and elevated HbA1c levels further contribute to kidney damage through mechanisms like increased glomerular pressure (32) and hyperglycemia-induced oxidative stress (33). As HbA1c is a key component of eGDR, and its values can be affected by anemia, particularly iron deficiency or chronic disease anemia, eGDR may be underestimated in such individuals. This potential bias warrants caution when interpreting eGDR in anemic populations. The observed interaction between eGDR and anemia status in our study further supports the need to consider hemoglobin levels when applying eGDR clinically. Our results show that eGDR has a stronger correlation with CKD indicators compared to HOMA-IR, QUICKI and TyG related indices. This could be due to eGDR's ability to integrate multiple metabolic risk factors, providing a more comprehensive and stable assessment of IR. The findings suggest that eGDR can serve as a valuable clinical tool, providing a straightforward and reliable method for early detection of CKD and risk stratification. Interestingly, we observed a significant interaction between eGDR and anemia status in relation to CKD risk. However, it is important to recognize that no single measure fully captures the complexity of insulin resistance and its relationship with CKD.

This study has several limitations that should be acknowledged. First, due to its cross-sectional design, causal relationships between insulin resistance markers and CKD cannot be established. Although we adjusted for multiple confounders, there remains the possibility of residual confounding from unmeasured variables such as genetic predisposition, dietary intake, and medication use. These factors could impact both insulin resistance and CKD risk, potentially influencing our findings. Additionally, some covariates, such as smoking status, alcohol consumption, and history of hypertension, were based on self-reported data, which introduces the possibility of recall bias and misclassification. Additionally, the study population is limited to a specific cohort, and further research is needed to validate these findings in diverse populations and settings. While the NHANES dataset provides a robust and nationally representative sample of U.S. adults, our findings may not fully generalize to populations outside of the U.S. or to individuals with more advanced stages of chronic kidney disease (CKD). The dataset's composition may not encompass all ethnic groups or clinical characteristics that could influence the relationship between insulin resistance and CKD.

To improve the generalizability of our findings, future studies should explore similar analyses in other diverse populations, including those from different geographical regions and those with more severe CKD stages. Additionally, examining the applicability of eGDR in cohorts with various comorbidities and risk profiles would be valuable for broader clinical use. Future research should focus on longitudinal studies to confirm the causal relationships between eGDR and CKD progression. While our study demonstrates a significant association between eGDR and CKD markers, the relatively low R² values suggest that other factors may better account for the observed variations in eGFR and UACR. The inclusion of additional variables in future models, such as genetic data, diet, and other biomarkers, could enhance the predictive power of insulin resistance indices. Expanding the scope of research to encompass additional confounding factors, including metabolic syndrome and non-alcoholic fatty liver disease, may elucidate the intricate interplay between insulin resistance and CKD more fully. We also recommend that future research explore alternative statistical approaches that can capture more complex relationships between eGDR and CKD.

## Conclusion

In conclusion, our study highlights the potential of eGDR as a practical and reliable marker for insulin resistance. It demonstrates significant associations with indicators of chronic kidney disease (CKD), underscoring its potential as an early detection and management tool. The simplicity of calculating eGDR, coupled with its robust predictive capabilities, positions it as a valuable asset in the clinical toolkit. Further research is warranted to explore its broader applications and confirm its utility in diverse clinical settings.

## Data Availability

The original contributions presented in the study are included in the article/**Supplementary Material**. Further inquiries can be directed to the corresponding authors.

## Supplementary material

Supplementary Figure 1 ROC curve analysis for eGDR, HOMA-IR, QUICKI and TyG-related indices in Predicting CKD stratified by diabetes status.
