# Supplementary figures and images for: Association of estimated glucose disposal rate with chronic kidney disease: comparative analysis against traditional insulin resistance indices
Source: Front Endocrinol (Lausanne). 2025 Jul 4;16:1507735. doi: 10.3389/fendo.2025.1507735 (PMC12272229; doi:10.3389/fendo.2025.1507735)

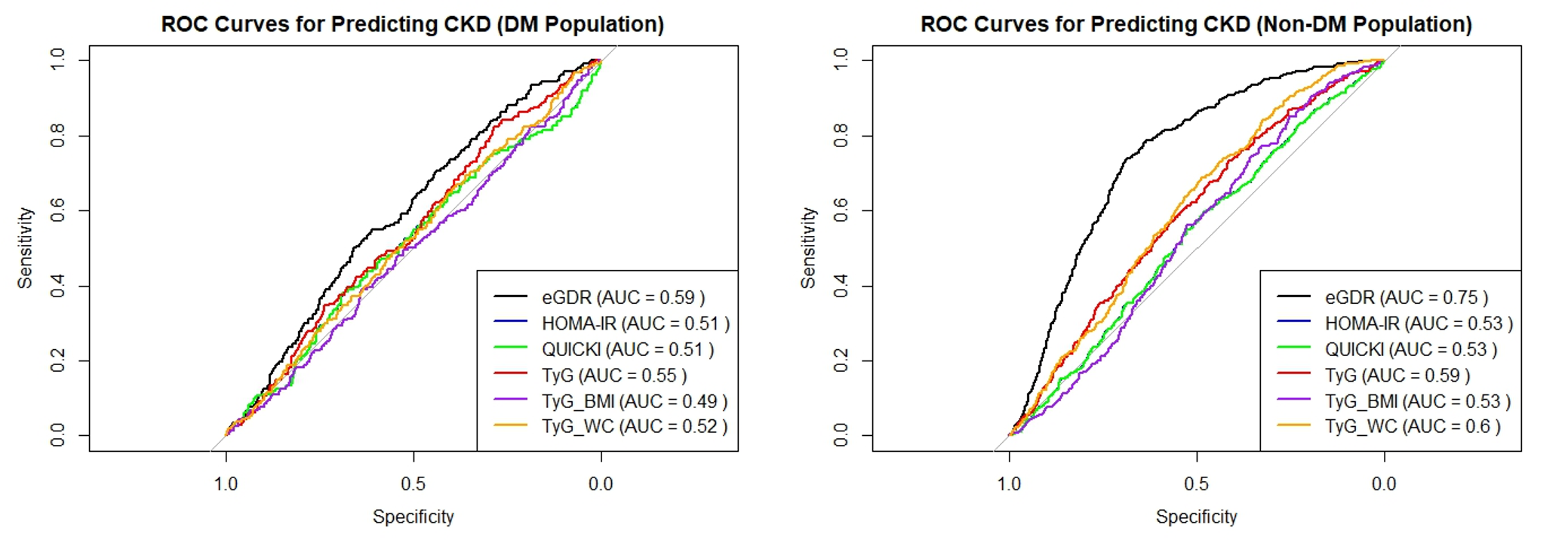

Supplement: Supplementary Figure 1 — ROC curve analysis for eGDR, HOMA-IR, QUICKI and TyG-related indices in Predicting CKD stratified by diabetes status. [file Image1.jpeg]
